# Standardization of Nitrous Oxid-Oxygen Anesthesia Induction*Read during the Sixth Annual Meeting of the Interstate Association of Anesthetists in Joint Session with the National Anesthesia Research Society and the Odontological Society of Western Pennsylvania, William Penn Hotel, Pittsburgh, October 5-7, 1920.—*Current Research*.

**Published:** 1921-08

**Authors:** J. A. Heidbring

**Affiliations:** Minneapolis, Minn.


### STANDARDIZATION OF NITROUS OXID-OXYGEN ANESTHESIA INDUCTION*

J. A. HEIDBRING, D.D.S., MINNEAPOLIS, MINN.

In early days before oxygen was used in combination, nitrous oxid, because of its fleeting qualities, was limited in its scope of usefulness. There was time only for short and hasty operations. When apparatus first appeared for combining oxygen the anesthesia could be prolonged, providing the anesthetist was sufficiently skillful to anticipate symptoms and make adjustments in the constantly varying mixture, due to the crude proportioning devices then obtainable. However, the addition of oxygen was a long step in the right direction.

Some became expert and administered beautiful anesthesia. Some were moderately successful. The majority failed miserably. There being no uniformity of dosage,

* Read during the Sixth Annual Meeting of the Interstate Association of Anesthetists in Joint Session with the National Anesthesia Research Society and the Odontological Society of Western Pennsylvania, William Penn Hotel, Pittsburgh, October 5-7, 1920.—*Current Research.*

there could be no uniformity of induction. The result was an exaggerated idea of the variation in susceptibility of individuals to nitrous oxid. These failures created in the minds of many the idea that there was something mysterious about nitrous oxid-oxygen administration, and that to be successful, one must possess the *sixth sense.* Much of this impression still persists and is the *bugaboo* standing in the way of many good and capable men beginning the use of nitrous oxid anesthesia. With present day apparatus, accurate predetermined dosage can be delivered and nitrous oxid-oxygen administration is *very* much simplified.

### THE NEED FOR CAPABLE ANESTHETISTS

There is a great need of more capable nitrous oxid-oxygen anesthetists, not only in the dental field, but in the field of general surgery, that humanity may benefit by this most valuable anesthetic.

Men of the professions in rapidly increasing numbers are beginning the use of nitrous oxid, many with insufficient preparation. These are dependent upon such as the members of this organization for their training. The burden is upon us. The more we can simplify and standardize technic, the more rapidly we can teach them, and the more will the public be safeguarded. To my knowledge there is little or no opportunity for this ever-increasing number to secure the prolonged training and experience, under supervision, necessary to make of them expert anesthetists. The best we can hope to do is to give them a foundation upon which to build, advising them to avoid the more difficult subjects until knowledge has been gained through experience.

### THE EVOLUTION OF STANDARDIZED INDUCTION

If it is practicable to simplify and standardize a technic for administering nitrous oxid-oxygen, necessitating the minimum deviation to accommodate exceptional cases or unusual conditions, then a long-felt want has been met.

With this idea in mind I conducted experiments, ending March, 1919, in approximately 4,500 cases, to determine the comparative susceptibility of patients. Since that date the findings have been verified by thousands of additional administrations both by myself and others.

A satisfactory dosage for inductions having been determined by experimentation, a definite technic for manipulating the apparatus was rigidly adhered to, and the anesthesia induction was timed by a stop watch. Then will all elements standardized, any variation in results was evidently due to variation in the susceptibility of the patient.

In selecting the dosage, the aim was to procure a comfortable induction and a distributed anesthesia in as short a time as practicable. Anesthesia should not be induced too rapidly. For instance, were we to administer pure nitrous oxid from the beginning, without dilution with oxygen or air, it acts so rapidly that the first nerve centers, with which it comes in contact, may become anesthetized before there was time for general distribution of the gas and the patient might show pronounced symptoms before complete anesthesia was actually accomplished. If operated upon, the patient might feel pain. Anesthesia so quickly induced is not well established, and is difficult to maintain while operating in the mouth.

### EXPERIMENTAL DATA

The dosage used in the experiments was nitrous oxid 95 per cent. and oxygen 5 per cent., administered for one minute, followed by pure nitrous oxid until the depth of anesthesia was obtained, usually indicated by the ashen hue of the face and slight twitching of the eyelids, which is the beginning of jactitation. Then oxygen was again added. All anesthesias were for oral operations and were carried to the stated depth to allow latitude for maintaining anesthesia with the mouth open. The dosage is not intended to apply to anesthesia for major surgical operations. For extra-oral operations, where both the mouth and nose may remain covered to exclude all air, a much lighter anesthesia can be easily maintained. Light anesthesia should be more slowly induced to allow time for better distribution of the gases.

Accurate records were kept of the induction of 2,044 consecutive anesthesias. Of this number 1,828 (or approximately 90 per cent.) responded uniformly to the common dose, were classed as *normal* and divided according to age as follows: 1,715 adults were anesthetized in 1 minute and 40 seconds; 43 children, aged 10 to 15 years, were anesthetized in 1 minute and 35 seconds; 70 children, aged 10 years or under, were anesthetized in 1 minute and 30 seconds.

The remaining 10 per cent. of patients required 5 to 20 seconds longer induction and were classed as *adnormal.* By observations made and the histories obtained they were divided into three types as follows: *Athletes,* 67 (robust individuals more resistent to anesthetic influence); *stimulated,* 113 (habitual users of alcohol or narcotics); *excitable,* 19 (the nervous or hysterical type). Seventeen apparently normal individuals varied from the common dosage as follows: 9 were anesthetized in 1 minute and 50 seconds; 3 in 1 minute and 35 seconds; 1 rapid breather was anesthetized in 1 minute and 35 seconds; 1 shallow breather and 1 shell-shocked soldier, who were anesthetized in 1 minute and 50 seconds; and 2 children, aged 10 to 15 years, anesthetized in 1 minute and 30 seconds.

### NORMAL AND ABNORMAL SUSCEPTIBILITY

*As previously stated it has been the general opinion that patients vary greatly in susceptibility to gas anesthesia, and that each one is a law unto himself. Abnormal patients, constituting 10 per cent., do vary from normal in susceptibility, some quite markedly; but nor-*

*mal individuals, constituting 90 per cent., are so nearly uniform in susceptibility that they respond quite similarly to a common dose.*

Do not understand me to recommend that the timing of the induction is to displace the observance of symptoms. The abnormals are to be reckoned with. Any variation in technic may also change the time of induction. A thorough knowledge of symptoms is imperative, and that they must, at all times, be observed and heeded is of paramount importance.

Timing furnishes the cue as to when anesthesia symptoms should appear. From the data given you will notice that in only three adults, or one in 700, was the time of induction less than one minute and forty seconds, and in these three cases the induction period was only five seconds less than normal. Doubtless the induction could have been prolonged five seconds without harmful results. Only one in ten required longer than normal induction.

### THE STANDARDIZED TECHNIC

It would seem practical then to instruct the beginner to follow a definite routine technic for manipulating his apparatus during the induction. With uniform dosage administered, anesthesia symptoms may be expected to appear at a definite time. In no case should the induction be carried beyond a certain time limit. It is exceedingly dangerous to attempt to put the extremely stimulated types of patients into *quiet* anesthesia. Using the described dosage, the time limit for induction has been set at two minutes.

When anesthesia is established 5 per cent. of oxygen is added and this mixture is increased in volume to force it through the nose. This amount of oxygen, plus the air usually inhaled through the mouth while operating, is sufficient to carry the majority of patients through an ordinary dental operation without necessitating changes in mixture or volume. When indicated, changes in oxygen percentage are made.

EVALUATING SYMPTOMS

The beginner usually finds difficulty in recognizing symptoms of complete anesthesia. Early appearance of any one symptom such as cyanosis, jactitation or stertor, is sometimes misleading. Cyanosis of some degree is a necessary accompaniment to nitrous oxid anesthesia. When nitrous oxid enters the blood stream it displaces oxygen. Therefore the blood is blue in proportion to the oxygen displaced. Since normal individuals when anesthetized have absorbed practically an equal amount of nitrous oxid, it is reasonable to presume that there is a corresponding uniformity of blueness of the blood. The extent to which this blueness is evident depends upon the complexion of the patient. Individuals with fair, thin skin, rosy cheeks or florid complexion, show the color of the blood more vividly and sometimes display marked cyanosis before completely anesthetized. Because of these premature symptoms the beginner often operates too soon and the patient suffers pain or developes excitement or both. The common error of beginners is to operate in an anesthesia too light to be maintained or to control the patient.

Timing assists in judging the maturity of symptoms.

Since conducting these experiments I have practiced and taught this method with good results. The beginner is furnished with a routine technic which he readily acquires.

The *experienced* anesthetist may have his own ideas as to what constitutes a desirable dosage or resulting anesthesia. But whatever his belief as to proportion of gases in the mixture, time for induction or depth of anesthesia, he will obtain more uniform results by following a standardized technic for the induction.—*Current Research.*

